# Genetic Variation, GWAS and Accuracy of Prediction for Host Resistance to *Sparicotyle chrysophrii* in Farmed Gilthead Sea Bream (*Sparus aurata*)

**DOI:** 10.3389/fgene.2020.594770

**Published:** 2020-12-22

**Authors:** Muhammad Luqman Aslam, Roberta Carraro, Anna Kristina Sonesson, Theodorus Meuwissen, Costas S. Tsigenopoulos, George Rigos, Luca Bargelloni, Konstantinos Tzokas

**Affiliations:** ^1^Nofima, Tromsø, Norway; ^2^University of Padova, Padua, Italy; ^3^Norwegian University of Life Sciences, Ås, Norway; ^4^Hellenic Centre for Marine Research, Heraklion, Greece; ^5^Andromeda Group, Rion Achaias, Greece

**Keywords:** single nucleotide polymorphisms, genomic selection, genome-wide association analysis, genetic correlation, 2b-RAD sequencing, genetic variation

## Abstract

Gilthead sea bream (*Sparus aurata*) belongs to a group of teleost which has high importance in Mediterranean aquaculture industry. However, industrial production is increasingly compromised by an elevated outbreak of diseases in sea cages, especially a disease caused by monogeneans parasite *Sparicotyle chrysophrii.* This parasite mainly colonizes gill tissues of host and causes considerable economical losses with mortality and reduction in growth. The aim of current study was to explore the genetics of host resistance against *S. chrysophrii* and investigate the potential for genomic selection to possibly accelerate genetic progress. To achieve the desired goals, a test population derived from the breeding nucleus of Andromeda Group was produced. This experimental population was established by crossing of parents mated in partial factorial crosses of ∼8 × 8 using 58 sires and 62 dams. The progeny obtained from this mating design was challenged with *S. chrysophrii* using a controllable cohabitation infection model. At the end of the challenge, fish were recorded for parasite count, and all the recorded fish were tissue sampled for genotyping by sequencing using 2b-RAD methodology. The initial (before challenge test) and the final body weight (after challenge test) of the fish were also recorded. The results obtained through the analysis of phenotypic records (*n* = 615) and the genotypic data (*n* = 841, 724 offspring and 117 parents) revealed that the resistance against this parasite is lowly heritable (*h*^2^ = 0.147 with pedigree and 0.137 with genomic information). We observed moderately favorable genetic correlation (*R*_*g*_ = −0.549 to −0.807) between production traits (i.e., body weight and specific growth rate) and parasite count, which signals a possibility of indirect selection. A locus at linkage group 17 was identified that surpassed chromosome-wide Bonferroni threshold which explained 22.68% of the total genetic variance, and might be playing role in producing genetic variation. The accuracy of prediction was improved by 8% with genomic information compared to pedigree.

## Introduction

Gilthead sea bream (*Sparus aurata*) is an economically important farmed fish species, specifically to the Mediterranean aquaculture industry with an annual production of ∼ 160,563 metric tons (FEAP Secretariat, 2017). The enhanced production and the demand of gilthead sea bream is also accompanied by production challenges with an increase of disease outbreaks (viral, bacterial, and parasitic) in sea cages (Food and Agriculture Organization of the United Nations [FAO], 2014), especially those which are caused by monogeneans parasites (Sitjà-Bobadilla, 2004; Nowak, 2007) with direct life cycles on the host. *Sparicotyle chrysophrii (S. chrysophrii)*, is a common monogeneans parasite which infests both wild and cultured gilthead seabream and instigates lethal epizootics in sea cages and is considered as one of the main diseases which threatens Mediterranean aquaculture. This parasite mainly colonizes gill tissues of the host and causes considerable economical losses with mortality ranging from 10 to 30% (Athanassopoulou et al., 2005; Muniesa et al., 2020), and, more importantly by reduction in growth of the farmed stocks triggered by emaciation and anemic state of the survivors (Sitjà-Bobadilla and Alvarez-Pellitero, 2009). Treatment of farmed fish against *S. chrysophrii* is very expensive, labor intensive, and requires resource demanding bath applications using anthelmintics which are also associated with environmental impacts (Basurco and Rogers, 2009; Rigos et al., 2013). Moreover, bath treatments may cause handling stress to the fish which may further increase losses.

Selection for genetic resistance against parasitic diseases is a highly valuable tool to help prevent or diminish disease outbreaks, especially where effective therapeutic agents or vaccines are limited or lacking (Bishop and Woolliams, 2014). The selection response is dependent on several factors including heritability, weight given to a trait, accuracy of selection and the generation interval. The pace of genetic progress can be increased through selective breeding (Ødegård et al., 2011) given that the trait is of moderate to high heritability (Odegård et al., 2014). Breeding programs in gilthead sea bream primarily included growth rate, and parasite/pathogen resistance with emphasis on minimizing productions costs (Chavanne et al., 2016; Janssen et al., 2017). Increasing the number of traits in the breeding goal could be complex and may impact selection response due to a reduced weight per trait and possible unfavorable genetic correlations among traits (Haffray et al., 2012; Yáñez et al., 2016).

The discovery of molecular markers [e.g., single nucleotide polymorphisms (SNPs)] and their associations with the traits of economic importance could prove very helpful to overcome unfavorable correlations among traits. Moreover, selection response in animal breeding programs especially for the traits which are difficult to improve (e.g., lowly heritable traits, meat quality traits, sex limited traits, etc.) by traditional selection can benefit from genome-wide distributed genetic markers and the knowledge about genetic markers linked to genes affecting quantitative traits (Meuwissen and Goddard, 1996; Pyasatian et al., 2007). The genetic markers have revealed significant associations with the quantitative traits of economic importance in Atlantic salmon (Everett and Seeb, 2014; Gonen et al., 2015; Moen et al., 2015).

Single nucleotide polymorphisms are popular molecular genetic markers widely used in aquaculture and livestock research (Gray et al., 2000; Aslam et al., 2012). Restriction-site associated DNA based sequencing (RAD-Seq) is a reduced representation high-throughput sequencing technique for the simultaneous detection and genotyping of individuals for the detected SNPs (Baird et al., 2008). The technique has been elaborated and used in several studies, briefly it employs (i) fragmentation of genomic DNA using restriction enzyme(s), (ii) selection of fragment size(s) from the digested DNA, unique individual specific nucleotide based barcoding and library preparation (iii) pooling the libraries from multiple samples, and high throughput sequencing. RAD-Seq technique has contributed in the development of genomic resources and studies on aquaculture species, i.e., discovery of SNPs, construction of high-density linkage maps (Kakioka et al., 2013; Palaiokostas et al., 2013a, b, 2015; Gonen et al., 2014) and genome wide association studies (GWAS) in a cost-efficient manner (Campbell et al., 2014; Palti et al., 2015; Tsai et al., 2016). A simplified version of RAD-Seq is known as 2b-RAD which requires type IIB restriction enzymes to digest genomic DNA from both ends (up and downstream) to the enzyme recognition site (Wang et al., 2012). The fragment size obtained with type IIB restriction enzymes are of uniform length (∼36 bp tags), and thus avoids the sampling error because size selection is not needed, which is required in RAD-Seq during the size selection step (Puritz et al., 2014). 2b-RAD approach has also been used and assessed by researchers for exploring genetic basis of traits in gilthead sea bream and other fish species (Fu et al., 2016; Palaiokostas et al., 2016; Pecoraro et al., 2016).

Moreover, there have been recent developments in genomic resources and tools for gilthead sea bream including the availability of a reference genome assembly (Pauletto et al., 2018; Pérez-Sánchez et al., 2019) and SNPs based genotyping array (Lozano, 2019). The development of these genomic resources/tools can also provide opportunities for advanced selection methods such as genomic selection. Genomic selection (GS) is an advanced method for predicted breeding values of candidates which utilizes the effects of genome-wide distributed markers potentially influencing quantitative traits with an objective to achieve reliable selection (Meuwissen et al., 2001). The use of GS becomes highly important for the traits which are difficult to measure on live selection candidates, e.g., carcass and disease resistance traits. The GS contribute through significant improvement in accuracy for predicting breeding values compared to accuracies obtained via traditional pedigree-based approaches (Palaiokostas et al., 2016; Tsai et al., 2016; Bangera et al., 2017; Vallejo et al., 2017).

The aim of current study was to explore genetic basis of host resistance to *S. chrysophrii* by estimating the role of genetic variation explaining resistance to this gill parasite, generate a linkage map, perform a GWAS to detect QTL, and finally compare accuracies of predictions based on genomic and pedigree data.

## Materials and Methods

### Resource Population

All the fish used originated from the breeding nucleus of the Andromeda Group which was developed with a cross of parents mated in ∼8 × 8 partial factorial crossbreeding design using 58 sires and 62 dams. The brooders of this species are known to be fluent spawners under natural photoperiod without any need of hormonal and/or light regimes. Parents were allowed to perform natural mass spawning events in eight different broodstock tanks. All the parents were tissue sampled for later parentage assignment and genomic analysis. The collection of fertilized eggs was performed in four consecutive days with equal number of eggs collected in each day from each of the eight tanks. The collected eggs were pooled each day to avoid any environmental variances. For this specific experiment, an average of around 250 fingerlings per tank (total *N* = 2,000) were randomly sampled from each tank and kept until they reached the average body weight of 16 g. The descendent fish taken from this mating design had approximately the same size.

### Challenge Test

A challenge test was performed following the controllable cohabitation infection model developed by Rigos et al. (2015) using donor fish as a prime source of disease transfer. The infection model is detailed and discussed in Rigos et al. (2015). In brief, 2,000 healthy gilthead sea bream weighing 16 ± 4 g were transferred to the facilities of the Hellenic Centre for Marine Research (Athens, Greece). Prior to the transfer, the fish were maintained in land tanks to avoid *S. chrysophrii* infections. Upon arrival, 20 fish were randomly examined by a stereoscopical analysis of their two external gill arches to ascertain the absence of the parasite. One thousand fish, considered as the recipient group, were transferred from the hosting tanks into a net cage (6 m^3^) located within an inland cement tank (50 m^3^). The remaining fish were kept in identical conditions in an adjacent cement tank and constituted the control group. One week later, 250 gilthead sea bream (50 ± 13 g) naturally infected with *S. chrysophrii* (infection confirmed by stereoscopy but level not counted) were introduced into the cement tank harboring the recipient fish and were left to swim freely outside of (around and beneath) the net for the whole experiment’s duration. With this design, donor fish were not interfering with the feeding environment of the recipient fish. Fish tanks were continuously supplied with aerated flow-through sea water. All fish were hand-fed a commercial diet at a daily rate of 2–2.5% of body weight. The whole experiment lasted for 10 weeks from the time when donor fish were introduced in the system until the final recording of gill parasites. The water temperature during the experimental period ranged from 27 to 20°C (summer-fall). At the beginning of the experiment, fish were weekly monitored (random sample of 10 fish) and subjected to stereoscopical examination until it was confirmed that the recipient fish had received the parasite. This sampling ended after 2 weeks at the second sampling event when the observed prevalence was found to be 100%.

### Traits

At the end of the experiment, all surviving recipients (*n* = 807) along with the control fish (*n* = 1,000) were individually weighed. The weighing of control fish was performed to evaluate the impact of infection vs. infection free environment on the growth. All recipient fish were slaughtered and subjected to stereoscopical examination (described above) for total counting of adult parasites of their 2 external gill arches. At the end of challenge test we had recordings on parasite count (PC), initial and final body weights (BW1 and BW2, weights at the beginning and at the end of the challenge test, respectively), and the specific growth rate (SGR) within the window (∼ 68 days) of the challenge test which was calculated using BW1 and BW2 information. The formula used for the calculations of SGR is given below.

SGR=(ln⁡BW⁢2-ln⁡BW⁢1)/68

Tissue samples of all the phenotyped fish were collected and stored in ethanol (100%) for further genomic work.

### DNA Isolation, Library Preparation and Sequencing

The genomic DNA was extracted from the collected tissue samples of ∼ 20 mg. The genomic DNA extraction method and library preparation protocol are detailed in Aslam et al. (2018a). In-short, 935 (120 parents and 815 juveniles) 2b-RAD libraries were constructed following the protocol reported by Wang et al. (2012) with minor modifications. The digestion of genomic DNA (300 ng) was performed using *AlfI* (Thermo Fisher Scientific, United States) enzyme. The digestion step was followed by ligation of library-specific adaptors, sample specific barcoding, and the enrichment of 2b-RAD fragments using PCR amplifications. The individual specific libraries were then pooled using two different multiplexing strategies for parents (64 libraries per pool) and offspring (128 libraries per pool) with an objective to have higher depth of sequencing in parents. The pooled libraries were quality controlled using Agilent 2100 Bioanalyzer, and finally sequencing of pooled libraries was performed on an Illumina NextSeq500 platform (Illumina, San Diego, CA, United States) with 50 base single-end sequencing (v2 chemistry, high output kit – 50 cycles).

### Genotyping 2b-RAD Loci

The genotyping from the individuals specific raw sequence reads was performed using following four steps,

(i) Adapter trimming and quality filtering was performed using custom developed scripts from the 2bRAD pipeline v2.0 (Wang et al., 2012). Poor quality reads were filtered out if mean Phred quality score within a sliding window of 4 bp was less than 15, and after the trimming and filtering, sequence reads of 36-bp length were kept.
(ii) The development of *de novo* reference sequence was performed by clustering of quality sequence reads of parents, which was accomplished with the use of Perl based scripts together with CD-HIT program (Fu et al., 2012; Wang et al., 2012).
(iii) Individual specific quality reads were then aligned to the developed reference sequence using bwa samse (*V* = 0.7.13-r1126; Li and Durbin, 2009) and the ‘mpileup’ function of SamTools version 1.2 (Li et al., 2009) was used to call variants and the call option of bcftools (Li et al., 2009) was used to call the genotype at each variant site for each fish.
(iv) The detection of reliable putative SNPs in the population was carried out using genotype quality (≥20), read depth (≥5), minor allele count (≥50) in the population at a particular site, and finally, minimum of 40 individuals were required to have genotype calls at the particular locus to fulfill mentioned criteria.

The SNP that passed the above-mentioned criteria were considered as a putative SNP for further analyses.

### Parentage Assignments

The genotype data was further filtered to obtain a panel of highly informative markers for parentage assignments. Hence, the SNP genotype data was pruned for both parents and offspring using minor allele frequency criteria (MAF ≥ 0.35). The filtering criteria retained 916 highly informative SNPs which were then used to construct the pedigree with the likelihood ratio method implemented in CERVUS version 3.0 (Kalinowski et al., 2007). The assignments obtained from likelihood ratio method were verified using the opposite homozygote count method (Hayes, 2011; Ferdosi et al., 2014) with the full set of SNPs.

### Genetic Linkage Mapping

The data was further pruned for the genetic linkage analysis, both at the marker and the individual levels. The filtering of markers was performed on, locus specific genotyping rate (≥70%), deviation from expected Mendelian segregation (*P* < 0.001), and the Hardy–Weinberg equilibrium exact test (*P* < 1.0 × 10^–7^). Filtering of individuals was performed using individual specific genotyping rate (≥70%). An additional criterion was used for building linkage only where individuals or families were removed if they had less than five full-sibs per family. This criterion was used to include informative families with sufficient information on recombination, and to avoid computational problems in building the map.

Linkage mapping was performed using Lep-Map v2 (Rastas et al., 2015) with the analysis steps described in Aslam et al. (2018a). Briefly, the module “SeparateChromosomes” was used with minimum LOD threshold value of 19 to obtain linkage groups, “JoinSingles” module was used at LOD score limit of 5 in combination with LOD score difference of 2, and finally the module “OrderMarkers” was used to estimate the order and distance between the markers in centiMorgans (cM). The linkage maps are reported as sex averaged maps unless otherwise indicated and map figures were plotted using R package LinkageMapView (Ouellette et al., 2018).

### Statistical Analyses

#### Descriptive Statistics

A generalized linear model was used in R to test the effects of recorded traits and to obtain initial evaluation of models. The growth traits (BW1, BW2, and SGR) did not show any significant effect on PC and therefore were not used as covariates. The initial body weight (BW1) showed a significant (*P* ≤ 0.001) effect on both SGR and BW2. The BW1 was not used as a covariate for BW2 to avoid genetic parameter deviations for BW2 due to potential collinearity between traits (BW1 and BW2) but it was used as a covariate for SGR, which should adjust for the same starting weight for all the fish and then obtain genetic variation for SGR. The raw data for PC presented a positively skewed distribution. Thus, to normalize this distribution a log transformation was performed on count values, i.e.,

$$\text{LPC}=\log_{e}\left(\text{PC}+1\right)$$

where LPC is a log transformed parasite count, and PC is the raw parasite count. The addition of 1 to PC was performed to avoid taking logarithm of 0 (since some fish had PC = 0).

#### Variance Components Estimation

The heritabilities and genetic correlations for LPC, SGR, and BW2 were estimated using ASReml 4.0 (Gilmour et al., 2015) with genomic and pedigree-based relationship matrices (G-matrix and A-matrix, respectively) using bivariate (LPC vs. SGR and LPC vs. BW2) mixed models.

(1) $$y_{\text{i}}=\mu +X\,b+Z\,u+e$$

where *y*_*i*_ is a vector of ‘*n*’ records on trait *i* (LPC, SGR, or BW2), μ, *b*, and *u* are overall mean, vector of fixed effects of BW1 applied on SGR only, and the vector of additive genetic effects, respectively. The vector of breeding values is assumed to follow either

*u*∼MVN(0,*G*⊗*G*_0_),or*u*∼MVN(0,*A*⊗*G*_0_), where *u* = $\left[\begin{array}{c} u_{1}\\ u_{2} \end{array}\right]$, $G_{0}=\left[\begin{array}{cc} \sigma_{u_{11}}^{2} & \sigma_{u_{12}}^{2}\\ \sigma_{u_{21}}^{2} & \sigma_{u_{22}}^{2} \end{array}\right]$, *G* = genomic relationship matrix, *A* = pedigree relationship matrix, *X* = incidence matrix for fixed effects, *Z* = incidence matrix for additive effects, and e is the vector of random residuals with *e*∼MVN(0,*I*⊗*R*_0_), where $R_{0}=\left[\begin{array}{cc} \sigma_{{\text{e}}_{1}}^{2} & 0\\ 0 & \sigma_{{\text{e}}_{2}}^{2} \end{array}\right]$.

The genomic relationship matrix was constructed using VanRaden (VanRaden, 2008) method 1 as $\frac{P\,P^{\prime }}{2^{*}\,\sum_{i=1}^{N\,s\,n\,p}p_{i}\,\left(1-p_{i}\right)}$; where *P*, *Nsnp*, and *p*_*i*_ are the matrix of centralized genotypes, total number of SNP markers, and the allele frequency of the reference allele, respectively. The narrow sense heritability was computed as the ratio of additive genetic variance to total phenotypic variance and genetic correlations were obtained from the above described bivariate analyses implemented in ASReml 4.0 (Gilmour et al., 2015).

#### Genome Wide Association Analysis (GWAS)

A univariate linear mixed model was used to perform genome wide association analysis for the LPC trait only. Model components were all the same as in model (1) except that the marker effects were also computed. The linear mixed model included random polygenic effects which accounted for family relationships (covariance between relatives) in the GWAS analysis. The marker – trait associations were performed using GCTA program (Yang et al., 2011) with the ‘–mlma-loco’ function which allows the estimation of a SNP effect by accounting the additive genetic variance expressed by all the markers distributed over all the linkage groups other than linkage group that contains the SNP.

The SNPs were considered as genome wide significant when they exceed the Bonferroni threshold for multiple testing *P*-value of *P* ≤ 2.29 × 10^–06^ with –log_10_(*P*) = 5.63, and if they surpassed *P*-value of *P* ≤ 5.74 × 10^–05^ with –log_10_(*P*) = 4.24 then they were graded as chromosome-wide significant. The significant values (chromosome and/or genome-wide) were computed as described in Aslam et al. (2020).

#### Quantile–Quantile (q–q) Plot

The q–q plot with the distribution of the observed –log_10_(*P*-values) for each SNP, and the expected –log_10_(*P*-values) from a theoretical distribution was plotted. The inflation factor (lambda, λ) was calculated using $\lambda =\frac{\text{meadian}\,\left(\chi^{2}\right)}{0.456}$, chi-square statisticsχ^2^ are obtained from significance-based *P*-values, and 0.456 is chi-square expected under the null hypothesis.

#### Estimation of the SNP(s) Variance

The proportional contribution of the chromosome and/or genome-wide significant SNP(s) to the total genetic variance was computed in ASReml 4.0 (Gilmour et al., 2015) as the reduction in the total genetic variance due to the addition of fixed effect of the SNP(s) in model (1). However, the *G* matrix used in this model was constructed with all other SNPs except the SNP(s) used as fixed effect in model (1).

#### Breeding Value Estimation

Breeding values were computed for LPC trait with univariate model (as described under the genome-wide association study) to quantify and compare accuracy of predictions obtained through pedigree (PBLUP) vs. genomic (GBLUP) information based BLUP models. The genomic relationship matrix was computed using all the quality markers (*n* = 21,773) left after filtering in genotyping process.

#### Accuracy of Prediction

For the estimation and the comparison of accuracies of prediction, we used records of individuals which had at-least one parent assigned and had genotype information along with the LPC record available. This limitation left us with 549 individuals available, and the accuracy of prediction for LPC was calculated as following.

A cross validation scheme was designed where ∼18% of the total 549 individuals were randomly masked. The random masking retained 449 individuals with information on LPC which were used as training set, while, randomly masked 100 individuals with missing phenotype records were used as validation animals. The breeding values were computed using genomic vs. pedigree relation matrices (*G*vs.*P*). The mean accuracy of 20 replicates was computed as correlation (*r*_*corr*_) of the estimated breeding value (pedigree/genomic) with the true phenotype, which were scaled by the square root of the heritability as $r_{\text{corr}}=\frac{\rho \,\left(G\,\left[P\right]\,E\,B\,V,y\right)}{\sqrt{h^{2}}}$; where ρ, *G*[*P*]*EBV*, *y*, and *h*^2^ are correlation coefficient, breeding values estimated using genomic or pedigree information, partially masked phenotypes, and genomic or pedigree based heritability estimates, respectively.

## Results

### Descriptive Statistics

The data statistics of recorded traits are presented in Table 1, together with the total number of observations for different traits (PC, LPC, BW1, BW2, SGR). There are some missing records due to some uncertain observation/counts. The distribution of parasite count and the log transformed parasite count is depicted in Supplementary File 1 (Supplementary Figures S1.1, S1.2).

**TABLE 1:** Descriptive statistics for the recorded growth and parasite count traits.

Traits	*N*	Missing values	Mean	Min	Max	SD
PC	807	192	5.75	0.00	45	5.88
LPC	807	192	1.57	0.00	3.83	0.85
BW1	807	1	17.19	2.00	60	8.96
BW2	807	192	43.93	11.60	87.8	11.65
SGR	807	192	0.016	–0.0012	0.033	0.0042

*N, number of records; min, minimum values; max, maximum values; SD, standard deviation; PC, parasite count; LPC, log transformed parasite count; BW1, body weight at the start of challenge test; BW2, at the end of challenge test: SGR, specific growth rate.*

### Genotyping RAD Alleles

Approximately 12 runs of sequencing for RAD libraries using on NextSeq 500 platform yielded a total of 4.271 billion reads. The generated total amount of sequence reads were distributed as 1.217 and 3.054 billion across parents and offspring, respectively. The mean number of raw reads for parents were 10.06 (±2.306) millions, while the offspring had low average mean raw reads with a value of 4.22 (±2.464) millions. The trimming and quality filtering step of raw reads slightly reduced the number with a loss of 74.5 million (0.17%) reads which resulted in an average number of 9.864 (±2.458) and 4.215 (±2.301) million quality reads available for parents and offspring, respectively.

The catalog which was developed from the quality reads of parents comprised of 269,660 unique loci, that was used as a reference sequence. The process of SNP calling discovered 33,684 2b-RAD tags which revealed at-least one SNP identified. The SNP data was further pruned based on MAF (≥0.02) and the locus specific genotyping rate (≥30%) with the aim of increasing overall informativeness and decreasing the amount of missing or erroneous information. This pruning step retained 21,773 quality SNPs for further analyses. Individuals were also filtered out if the individual specific rate of genotyping was < 30%, which resulted to a drop of 94 individuals (91 offspring + 3 parents), and ultimately SNPs based genotype data comprised of 21,773 loci typed on 841 individuals (724 offspring 117 parents).

### Parentage Assignments

The analysis of parentage assignments revealed 154 full-sib families with 1–22 sibs per family (Supplementary File 1 and Supplementary Figure S1.2). Out of the 724 offspring, 659 got both parents assigned, 62 got assignment of a single parent (sire or dam), while 3 offspring did not get any assignment. The validation of parentage assignment results using likelihood vs. opposite homozygotes count methods revealed 95% concordance. Out of the total 154 full-sib families, 45 families were used for the construction of linkage mapping which had a minimum of five sibs per family.

### Linkage Map

The process of linkage mapping using 21,773 SNP markers in total, generated a linkage map comprised of 15,184 SNPs which were grouped into 24 linkage groups (*SA01-SA24*). The remaining 6,589 SNP markers did not get assignments to any group. The obtained total sex average map length was 1406.02 cM (Table 2, Supplementary Figure S1.3, and Supplementary File 2). The highest number of markers was found in linkage group *SA02* with 754 markers, while the lowest number of markers were found in linkage group *SA24* with 496 markers. The number of SNPs and the corresponding chromosome map length showed an average (for 24 linkage groups) correlation of 0.412. The sex specific maps (male vs. female) showed that the female genetic map length is larger than the males with total map length of 1,598.19 and 1,236.35 cM, respectively.

**TABLE 2:** The genetic map of gilthead sea bream, *Sparus aurata (SA)* with sex specific and average map information.

Linkage groups	Number of markers	Male map length (cM)	Female map length (cM)	Average map length (cM)	CHR	Physical map length (Mb)
*SA01*	710	55.44	62.71	57.75	CHR02	25.58
*SA02*	754	65.70	74.91	70.83	CHR03	23.15
*SA03*	639	59.83	60.82	59.69	CHR05	23.49
*SA04*	681	33.89	68.39	51.03	CHR06	25.96
*SA05*	744	48.74	73.79	61.12	CHR01	24.95
*SA06*	642	53.30	67.07	60.39	CHR23	20.46
*SA07*	715	81.19	81.97	80.97	CHR13	22.66
*SA08*	661	32.68	55.03	43.37	CHR04	25.32
*SA09*	649	73.21	75.09	72.78	CHR21	20.82
*SA10*	630	44.21	67.02	54.22	CHR17	24.40
*SA11*	649	58.26	80.85	69.20	CHR11	23.14
*SA12*	594	52.79	61.15	54.54	CHR08	24.21
*SA13*	619	26.17	60.60	43.13	CHR07	25.41
*SA14*	603	62.05	64.88	63.86	CHR12	20.78
*SA15*	701	61.72	67.14	63.71	CHR10	17.45
*SA16*	614	23.80	57.72	40.02	CHR09	23.07
*SA17*	622	53.92	71.62	62.98	CHR15	22.26
*SA18*	613	54.90	55.97	54.32	CHR19	20.54
*SA19*	575	49.93	63.26	56.61	CHR20	20.43
*SA20*	581	57.17	67.12	61.83	CHR22	20.22
*SA21*	580	58.93	61.93	60.56	CHR14	19.05
*SA22*	600	53.69	69.42	61.09	CHR16	20.49
*SA23*	512	22.84	77.52	50.14	CHR18	21.73
*SA24*	496	52.00	52.21	51.87	CHR24	15.90
Unknown	6,589	–	–	–	–	–
Total	21,773	1,236.35	1,598.19	1,406.02	–	531.47

*CHR, The corresponding chromosome in the genome (Saurata_v1) to the linkage map; cM, linkage map length in centiMorgans; Mb, Physical map length in million bases covered by linkage map, the length (Mb) from the first to the last marker of linkage map.*

The loci of assigned linkage groups were examined for their corresponding positions and order on physical map using alignment of tags to the genome assembly, Saurata_v1 (Pauletto et al., 2018). The results revealed more than 90% concordance in order of marker with coverage of each chromosome shown in Table 2. Moreover, linkage disequilibrium (LD) across markers from each linkage group were plotted using genotype data for parents only to present the distribution of markers along with the pattern of LD decay with increasing marker distances. The pattern showed decreasing trend of LD with increasing marker distances and the distribution of markers display the coverage of each chromosome (Table 2, Supplementary File 1, and Supplementary Figure S1.4).

### Estimates of Variance Components and GWAS

The heritability estimates of LPC, SGR, and BW2 were very similar across the models (univariate vs. bivariate) and information sources (genomic vs. pedigree). The analysis using pedigree information resulted in heritability estimates of 0.147 ± 0.069, 0.155 ± 0.068, and 0.169 ± 0.072, for LPC, SGR, and BW2, respectively. Heritability estimates obtained with genomic information were 0.137 ± 0.061, 0.229 ± 0.077, 0.226 ± 0.074 for LPC, SGR, and BW2, respectively. The LPC trait showed a strong favorable negative genetic correlation of −0.807 ± 0.242 and −0.769 ± 0.224 (pedigree and genomic, respectively) with the SGR while −0.549 ± 0.269 and −0.701 ± 0.208 (pedigree and genomic, respectively) with BW2 (Table 3).

**TABLE 3:** Estimates of variance components for PC and SGR using model 1 with pedigree vs. genomic information.

LPC vs. (SGR and BW2)		
Components	Pedigree	Genomic
LPC $\left(\sigma_{\text{u}}^{2}\right)$	0.102 (0.050)	0.097 (0.045)
LPC (*h*^2^)	0.147 (0.069)	0.137 (0.061)
SGR $\left(\sigma_{\text{u}}^{2}\right)$	1.14e−06 (5.3e−07)	6.0e−06 (1.0e−06)
SGR (*h*^2^)	0.155 (0.068)	0.229 (0.077)
Genetic correlation (*R*_*g(LPC, SGR)*_)	−0.807 (0.2418)	−0.769 (0.224)
BW2 $\left(\sigma_{\text{u}}^{2}\right)$	23.730 (10.54)	32.076 (11.479)
BW2 (*h*^2^)	0.169 (0.072)	0.226 (0.074)
Genetic correlation (*R*_*g(LPC, BW2)*_)	−0.549 (0.269)	−0.701 (0.208)

*LPC, log transformed parasite count; SGR, Specific growth rate; BW2, body weight at the end of challenge test; $\sigma_{\text{u}}^{2}$, additive genetic variance; $\sigma_{\text{p}}^{2}$, phenotypic variance; h^2^, heritability; R_*g*_, genetic correlation.*

**FIGURE 1:**
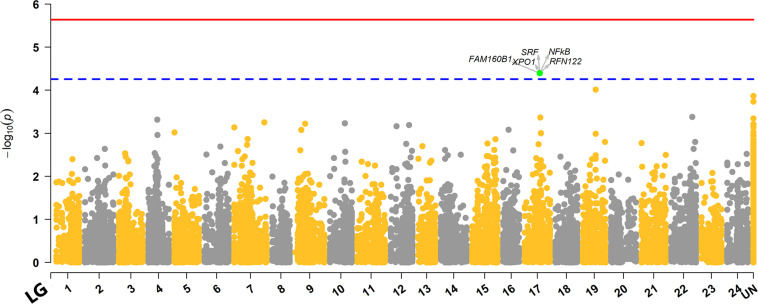
Manhattan plot for distribution of *P*-values across different linkage groups. The unmapped markers are annotated as “UN” group in the plot. The red solid line represents the genome-wide while the blue dashed line displays the chromosome-wide significant Bonferroni thresholds. The SNP that crossed chromosome-wide Bonferroni threshold is highlighted green, and genes within ± 100Kb region of the top significant SNP are highlighted with arrows.

The genome-wide association analysis revealed a single SNP at linkage group SA17 which crossed the Bonferroni-corrected chromosome-wide significance threshold with a *P*-value of 4.02 × 10^–5^ and allele substitution effect **α** of −0.374 for LPC (Figure 1 and Table 4). The allele substitution effects, and the minor allele frequencies for the top 5 SNPs are presented in Table 4. The proportion of the total genetic variance explained by the highest significant SNP was 22.68% estimated using the top SNP as fixed effect in the model.

**TABLE 4:** The statistics of the top 5 significantly associated SNPs with effect sizes.

SA	Locus ID	Pos (cM)	Allele1	Allele2	MAF	α ± se	*P*-value
17	116287_33	37.45	A	B	0.136	−0.3740.091	4.02e−05
19	19368_20	31.65	B	A	0.081	0.3820.098	9.94e−05
Un	39140_5	0	B	A	0.059	0.4590.121	1.39e−04
Un	70234_2	0	B	A	0.063	0.4290.112	1.87e−04
17	97289_18	38.91	B	A	0.054	−0.4350.123	4.31e−04

*SA, linkage group number for the gilthead seabream (Sparus aurata, SA), and the markers which did not group into any linkage group are represented by Un; Pos (cM), genetic map position of SNP; Allele1 and Allele2, minor and major alleles, respectively; MAF, minor allele frequency; α ± se, allele substitution effect with standard errors; P, significance value.*

#### Quantile–Quantile Plot

**FIGURE 2:**
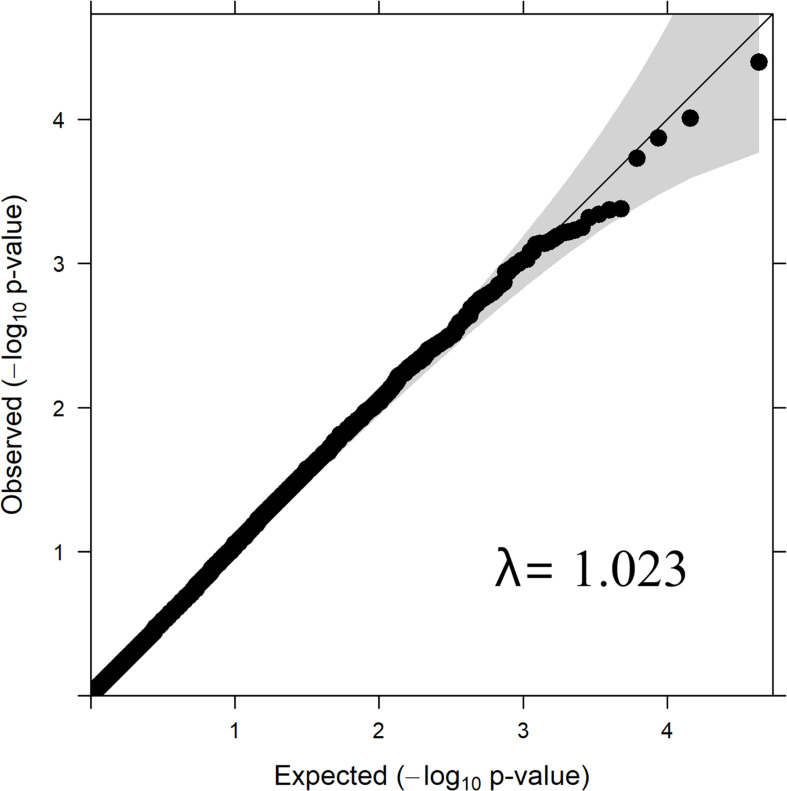
QQ-Plot of *P*-values – Log Parasite Count.

Quantile–quantile plot with the distribution of observed vs. expected *P*-values is depicted in Figure 2. The genomic inflation factor (lambda, λ) is a genomic control to get an estimate on false positives. The obtained λ-value from the fitted GWAS model with all markers was 1.023 (Figure 2). The observed *P*-values did not seem elevated in the q-q plot, which suggest that the GWAS analysis was not suffering from some unknown structure in the data, and thus also not from spurious associations. This is also confirmed by the value of λ= 1.023, which is close to 1.

### Accuracy of Prediction

Prediction accuracies obtained using genomic information was 0.55 ± 0.165 which was higher than the accuracies obtained using pedigree information with estimates of 0.51 ± 0.147.

## Discussion

*Sparicotyle chrysophrii*, a monogeneans gill parasite is one of the most severe threats for sea-cage production of gilthead sea bream in the Mediterranean. The infestation by *S. chrysophrii* affects all age groups in the sea which not only cause poor growth of fish but also mortalities that can go as high as 30% (Muniesa et al., 2020). The mortality at later stages when the fish is at the market size is likely to cause much more economic loss to the farmers. The treatment against this parasite includes chemicals such as formalin, hydrogen peroxide, and praziquantel which are very effective but are considered environment unfriendly and unsafe. Moreover, these applied available treatments affect consumer’s perception on product quality, ethics of production especially when there are increasing demands toward ecological products. The treatment cost against *S. chrysophrii* can go substantially high (€∼0.3 per kg fish produced) and the stocks may require multiple treatments in a production cycle. The cost of treatment for amoebic gill disease, which is caused by amoebic parasite, *Paramoeba perurans* accounts for 10–20% of the production cost in Australian farmed A. salmon (Nowak, 2012). Hence, treatments are not long-term remedy toward sustainable production. However, genetic improvement of commercial bream populations for resistance against *S. chrysophrii* (defined as low parasite count) using advanced selection methods may prove to be very useful, effective and preferred strategy especially when the trait is lowly heritable. Upgrading the overall robustness or resistance level against pathogens and/or parasites would avoid treatments using hazardous chemicals which should ultimately reduce environmental risks and promote sustainable production.

In the current study, we found significant difference in average weight (BW2) of challenged vs. control fish which was 43.93 g (SD = 11) and 58.01g (SD = 12), respectively, which suggests that the *S. chrysophrii* leads to a poor welfare of fish with effects on growth. Limited information is available for genetic variation of host resistance against *S. chrysophrii* parasite. However, there are several studies on genetic variation for resistance against different ectoparasites in Atlantic salmon and livestock species. In Atlantic salmon, resistance against amoebic gill disease where amoebic parasite colonizes the gill tissues and causes distress for the host is also reported to have a moderate heritability (Taylor et al., 2007; Kube et al., 2012; Robledo et al., 2018; Lillehammer et al., 2019). Similar estimates were obtained for host resistance to sea lice; ∼0.2 to 0.3 for the North Atlantic sea louse (Gjerde et al., 2011; Tsai et al., 2016), and 0.1–0.3 for the Pacific sea louse (Yáñez et al., 2014; Correa et al., 2017). Likewise, heritability estimates reported for resistance to common ticks in cattle were moderate (Shyma et al., 2015). Heritability estimates for resistance against *S. chrysophrii* gill parasite, i.e., LPC obtained in our study were low (0.147 and 0.137 with pedigree vs. genomic information, respectively) but significant, suggesting that the selection for improved resistance could be affective.

The analysis of genetic correlations showed favorable associations of LPC with both growth traits, i.e., SGR and BW2 (−0.549 to −0.807, respectively), depicting the decreasing trend of parasite counts toward heavier and fast growing families. Favorable relations among the traits should allow selection for growth and resistance to be performed simultaneously. Moreover, selection for growth should help to get correlated response for increasing resistance against *S. chrysophrii.* A similar favorable genetic correlation (–0.88) has been described for the severity gill disease due to *Paramoeba perurans* (gill parasite) and the body weight in Atlantic salmon (Gjerde et al., 2019). The reported genetic correlations for lice counts and growth traits have also been either close to zero or slightly favorable in Atlantic salmon (Tsai et al., 2016).

The current study produced a medium-density linkage map comprising 15,184 SNP markers distributed over 24 linkage groups. The generated numbers of linkage groups are consistent with the number of chromosomes/karyotype of gilthead seabream (Cataudella et al., 1980; Pauletto et al., 2018). The linkage map length in our study was 1,406.02 cM, which is shorter than what was obtained in previous studies by Tsigenopoulos et al. (2014) and Aslam et al. (2018a), where the sex-average map length was reported to be 1,970.29 and 3,899 cM, respectively. The difference in map lengths could also be explained by the difference in genome coverage, map resolution and population differences. However, the linkage map length reported by Palaiokostas et al. (2016) was ∼2.77 times longer than the map length obtained in the current study, which could be due to differences in adopted parameters and methods. Conversely, it was also observed that the high recombination rates involving false recombination detected due to data issues (e.g., genotyping errors, null alleles and nonrandom missing genotypes) may also cause the inflated genetic lengths (Jorgenson et al., 2005; Ronin et al., 2012).

Sex biased recombination has been observed in many fish species including Atlantic salmon, rainbow trout, zebrafish, etc., with significant reduction in recombination rate in heterogametic sex (Sakamoto et al., 2000; Singer et al., 2002; Moen et al., 2008). Mammal species, e.g., human, dog, pigs, etc. have also shown similar trend (Broman et al., 1998; Wong et al., 2010; Tortereau et al., 2012) where heterogametic male expressed lower recombination rates than the female. However, gilthead sea bream is a sequential protandrous hermaphrodite with majority of fish being able to produce either kind of gamete (sperm or ova) at different stages of their life. The *S. aurata* individuals in their first reproductive cycle mature as males (at the age of 2 years), though ovaries start differentiating in the larval stage but are replaced by the testes (Mylonas et al., 2011) as fish matures at 2 years of age, and then in the subsequent cycles, the testes regress and functional ovary develops in some males. Regardless of the hermaphrodite nature, we observed sex biased recombination in *S. aurata* with female-to-male recombination rates of 1.29:1.0 (Table 2) and the total female-specific map was 361.84 cM longer than the male specific map. The results obtained from our study on heterochiasmy were concordant with other studies (Franch et al., 2006; Tsigenopoulos et al., 2014; Aslam et al., 2018a). However, Palaiokostas et al. (2016) did not observe much difference in male vs. female recombination (1:1.05, female-to-male recombination rate) with a male specific map (4,010 cM) being slightly longer than the female map (3,822 cM). This discordance could be due to population specific differences, and/or different methods used for constructing the linkage maps. There are several other reports on the existence of heterochiasmy in hermaphroditic animals where females have shown a higher recombination rate than males (Theodosiou et al., 2016). The phenomenon of heterochiasmy in hermaphroditic species has been argued to be an effect of the female meiotic drive, which is considered as a mechanism in the evolution of neo-sex chromosomes (Yoshida and Kitano, 2012; Theodosiou et al., 2016; Abbott et al., 2017).

Our genome wide association analysis revealed no major QTL region(s) that reached the genome-wide significance threshold for LPC (Figure 1). However, there was a signal for a suggestive QTL with one SNP detected on linkage group 17 (*SA17*) that crossed the chromosome-wide Bonferroni threshold (Figure 1). This chromosome-wide significant SNP had a MAF of 0.136 with an allele substitution effect (α) of -0.374 in favorable direction (Table 4), which means that the substitution of allele should have a parasite reducing effect. Though, the second SNP of *SA17* (Table 4) had low MAF (0.054) but showed an α value of similar magnitude to the top significant SNP of *SA17* (Table 4). Three other SNPs, one from *SA19* and the two from unknown (Un) linkage group also showed strong significance *P*-value but did not cross the threshold line (Figure 1 and Table 4). These SNPs and possibly multiple other loci may have crossed the threshold significance line with increased power (using more individuals with phenotype information) given that the association of these SNPs are not merely by random chance. As, these SNPs are distributed across different linkage groups and have shown some level of effect on trait, though, only one SNP is crossing suggestive chromosome-wide level of significance. From these results, one could argue about polygenic nature of trait with possibility of a few loci with relatively large and many loci with potentially small effects. In Atlantic salmon, resistance against parasitic diseases such as amoebic gill disease and sea lice resistance have also been reported to be very polygenic in nature (Tsai et al., 2016; Robledo et al., 2018). We detected a slight LD of 0.15 between the two markers of linkage group *SA17* (Supplementary Figure S1.5). However, markers of unknown groups in Table 4 expressed relatively higher LD of 0.33 with each other but showed close to zero LD with markers of *SA17* and *SA19*. The LD information among markers of different groups of Table 4 further strengthens the hypothesis of a polygenic nature of LPC, as close to zero LD for the markers of different linkage groups exempts the chance of misplacement of these markers during linkage mapping.

The λ value obtained from the *P*-values of SNPs from GWAS analysis/model was 1.023 (Figure 1), which was close to 1.0 indicating that the *P*-values are not inflated by any population stratification. Hence, no adjustments to the model was required when lambda is less than or equal to 1.0, as explained by Hinrichs et al. (2009).

The observed reduction in genetic variance was 0.022 (0.097–0.075) when an additional fixed effect of chromosome-wide significant SNP (Locus ID: 116287_33) was used in model 1. Hence, the proportion of genetic variance explained by the SNP was 22.68% which seems quite high compared to the QTL signal in the Manhattan plot (Figure 1). The tagged SNP (116287_33) might be highlighting an important QTL region explaining variation either directly or through LD with the true QTL. However, a seemingly inflated high proportion of genetic variation explained by the SNP could be a result of the Winner’s curse (Nakaoka and Inoue, 2010). We had only 549 recorded individuals with genotypes available for this complex parasite resistance trait and the top significant SNP (116287_33) did not pass the genome-wide threshold and therefore a validation of this QTL in an independent population is needed. The concordance of the results from validation study would signify the importance of the discovered QTL region and improve confidence for possibly performing efficient and economical QTL based selection for the industry. Moreover, the top 5 SNP markers of the current study that presented large effect sizes had relatively low MAF, which signifies the importance of rare mutations as explained in studies from Dickson et al. (2010) and Eichler et al. (2010) on synthetic associations and missing heritability due to rare mutations.

Cellular and molecular mechanisms underlying the host response to *S. chrysophrii* infection remain largely unknown (Sitjà-Bobadilla and Alvarez-Pellitero, 2009; Henry et al., 2015). Long-term infection apparently induces severe anemia, lower humoral response and impaired innate immune response (higher respiratory burst, but lower antimicrobial activities). The tag sequence (36 bp) of the highest significantly associated tag/SNP (116287_33) was aligned to the sea bream genome assembly, Saurata_v1 (Pauletto et al., 2018) to potentially obtain molecular insight which might help to understand possible causes in trait variation. The tag sequence aligned to chromosome 15 with a significant *e*-value = 4.00 × 10^–9^. The region of approximately ± 100 Kb surrounding the highest significant SNP “116287_33” was searched for the underlying candidate genes which resulted in five genes (*RFN122, XPO1, NFkB, SRF*, and *FAM160B1)* encompassed in the candidate genomic region that have been reported to be involved in immune response in other species. *RFN122* suppresses antiviral type I interferon (Wang et al., 2016) in virus-infected cells. Its potential role in the infection of an ectoparasite, however, needs to be determined. *Exportin-1* (*XPO1 or CRM1*) is a nuclear export receptor involved in the transport of specific mRNAs. Inhibition of *XPO1* was reported to down-regulated dendritic cell (DC) maturation (Chemnitz et al., 2010). As DCs are the most potent antigen-presenting cells, *XPO1* differential expression/activity due to genetic variants might have broad effects on immune response. More generally, *XPO1* has been shown to be involved in the regulation of *NFkB*, a key transcription factor in cell survival and immunity. *Neurixn-1a* is a single-pass type I membrane protein that is mainly expressed in the nervous system and there is no evidence so far of its involvement in immune function. Alternatively, *serum response factor* (*SRF*) is involved in many aspects of the immune system. Particularly relevant is *SRF’s* role in hematopoiesis and neutrophil migration during inflammation (Taylor and Halene, 2015), considering that in sea bream exposed to *S. chrysophrii*, increased activity of *myeloperoxidase* (*MPO*) was observed (Henry et al., 2015). Activity of MPO generally correlates well with levels of inflammatory responses sustained by neutrophilic granulocytes. Therefore, it might be possible that individual variation in *SFR* function has an effect on host-response to the parasite. Finally, *FAM160B1* is a poorly characterized protein with no known involvement in immune response.

The accuracy of prediction obtained for LPC trait was fairly high with accuracy of 0.51 with pedigree and 0.55 using genomic information. Comparison of accuracies of predicting breeding values using PBLUP vs. GBLUP clearly shows that the genomic information based predictions are more accurate. Overall, we observed an ∼8% increase in accuracy with genomic information reflecting an expected advantage in genetic gain with genomic information. The complicated parasite resistance traits (e.g., lice and amoebic gill disease) in Atlantic salmon have also shown a similar trend with advantages of genomic over pedigree based predictions (Tsai et al., 2016; Robledo et al., 2018). The advantage of GBLUP over PBLUP is due to the fact that realized genomic-based relatedness between animals deviates from pedigree-based relationship coefficients. In addition, genomic breeding values are not affected by pedigree errors, though they can be affected by genotyping errors or introduction of mistakes during sample identifications. The current study had only rather shallow pedigree data available which might also have been a slight disadvantage for PBLUP.

The GS becomes even more beneficial compared to PBLUP when the trait is complex, difficult to record, lowly heritable and not recorded on the selection candidates. Additionally, GS can improve genetic gains through improving the accuracy of breeding values, and better use of variation within family (García-Ruiz et al., 2016; Zenger et al., 2019). Hence, in current era of genomics, the application of genomic selection is unavoidable in order to stay in industrial competition, to produce efficiently, and to follow market demand. The increase in accuracy of predictions (∼8%) for parasite count using genomics seem marginal with respect to the potential expenses on genotyping costs. However, the trait specific expected economic gain is very much dependent on the amount of production, production environment, given weight to the trait, and adopted strategy to develop production stock.

## Conclusion

The current study shows that the 2b-RAD approach for the detection of SNPs and for the genotyping of a sea bream population is an effective method which resulted quality genotype data for ∼21K SNPs. The analysis reveals that the resistance against *S. chrysophrii* is significantly heritable (0.147 ± 0.069) and therefore genetic improvement is a valuable tool to reduce the prevalence of *S. chrysophrii* in farmed gilthead sea bream. The genetic architecture for host resistance to *S. chrysophrii* appears to be polygenic with one suggestive QTL detected at linkage group 17 which explains a large proportion (22.68%) of genetic variance. Since this SNP did not pass the genome-wide threshold, a validation of this QTL in an independent population is needed. The accuracy of predicting breeding values with genomic information was substantially higher (∼8%) than predictions using pedigree information. *S. chrysophrii* is a significant and increasing threat for Mediterranean sea bream production, and implementation of genomic selection in breeding programs may prove an efficient methodology to genetically improve host resistance to this parasite.

## Data Availability Statement

Most of the data supporting the results are included within the manuscript as additional files. However, due to competitive nature and the privacy policy of commercial breeding companies, it is not permitted to share phenotypic, sequence, and genotypic data. The data can be made available for specific queries (on agreement) through the corresponding author.

## Ethics Statement

The animal study was reviewed and approved by local ethical committee for the use of animals in the study. The reference code for the acquired approval is EL 25 BIO 37. Written informed consent was obtained from the owners for the participation of their animals in this study.

## Author Contributions

MA analyzed the data and drafted the manuscript. RC performed the 2b-RAD library preparations and the other molecular laboratory-based analyses. AS, TM, CT, GR, LB, and KT were involved in the design of study, sampling, phenotype recording, and actively contributed in discussions. KT also assisted in arranging population material. KT, LB, CT, and GR played role in collection of data and coordination at the testing facility. AS and TM assisted in statistical data analysis. All authors read the manuscript, gave suggestions and comments for the improvement, and approved the final manuscript.

## Conflict of Interest

The authors declare that the research was conducted in the absence of any commercial or financial relationships that could be construed as a potential conflict of interest.

## Supplementary Material

Supplementary File 1 Supporting information on results including information on parasite count trait, density of markers across different linkage groups and LD among markers.

Supplementary File 2 The order of 2b-RAD tags on linkage map along with SNP positions and tag sequences.

## Abbreviations

LPC: log transformed parasite count
*h*^2^: heritability
SGR: specific growth rate
BW: body weight
GS: genomic selection
QTL: quantitative trait locus
SNPs: single nucleotide polymorphisms
RAD-Seq: restriction-site associated DNA sequencing
GWAS: genome wide association studies
QC: quality controls
LG: linkage groups
cM: centiMorgans.
