# Supplementary material for: Genetic Variation, GWAS and Accuracy of Prediction for Host Resistance to Sparicotyle chrysophrii in Farmed Gilthead Sea Bream (Sparus aurata)
Source: Front Genet. 2020 Dec 22;11:594770. doi: 10.3389/fgene.2020.594770 (PMC7793675; doi:10.3389/fgene.2020.594770)
Supplement: Supplementary file 2 [file Data_Sheet_1.docx]

**Genetic basis of host resistance to *Sparicotyle chrysophrii* in farmed gilthead sea bream (*Sparus aurata*) population**

*Muhammad Luqman Aslam ^*^****^1^****, Roberta Carraro* ***^2^****, Anna Kristina Sonesson* ***^1^****, Theodorus Meuwissen ^3^, Costas. S. Tsigenopoulos****^4^****, George Rigos****^4^****, Luca Bargelloni* ***^2^*** *and Konstantinos Tzokas^5^*

***^1^*** *Nofima, P.O. Box 210, N-1431 Ås, Norway*

***^2^*** *University of Padova, 35020 Legnaro, Italy*

***^3^*** *Norwegian University of Life Sciences, Norway*

***^4^****Hellenic Centre for Marine Research (HCMR), Heraklion, 71500, Greece*

***^5^****Andromeda Group, 26504 Rion Achaias, Greece*

**Figure S1.1:** Distribution of parasite count and the log transformed parasite count phenotypes.


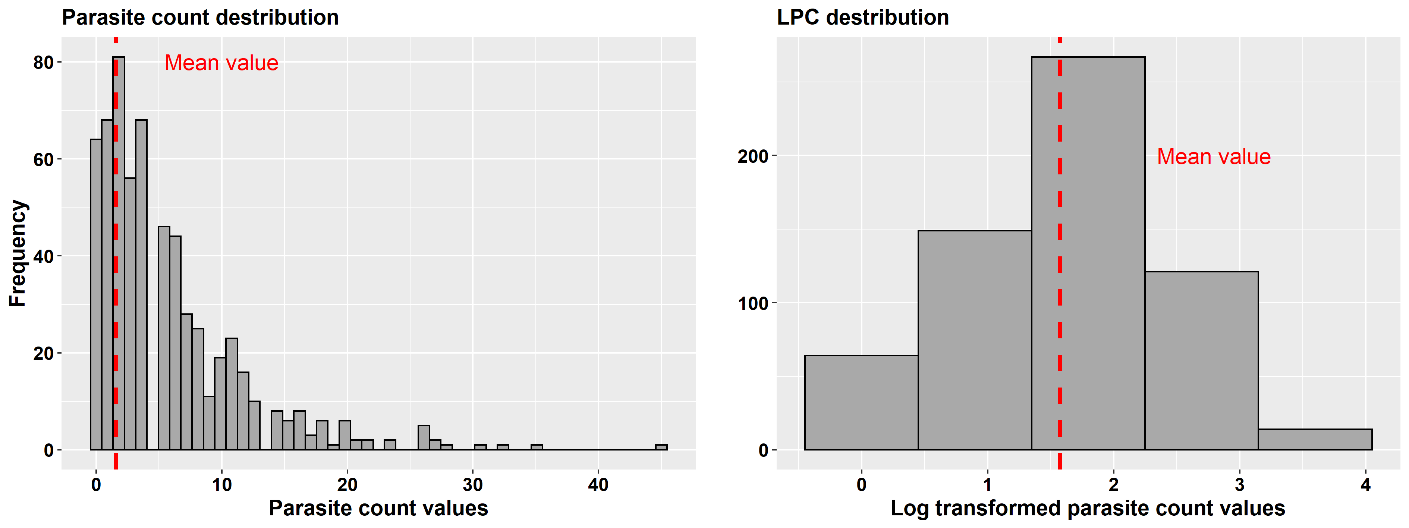


**Figure S1.2:** Distribution of number of sibs per family along with the distribution of phenotype across full-sib families.


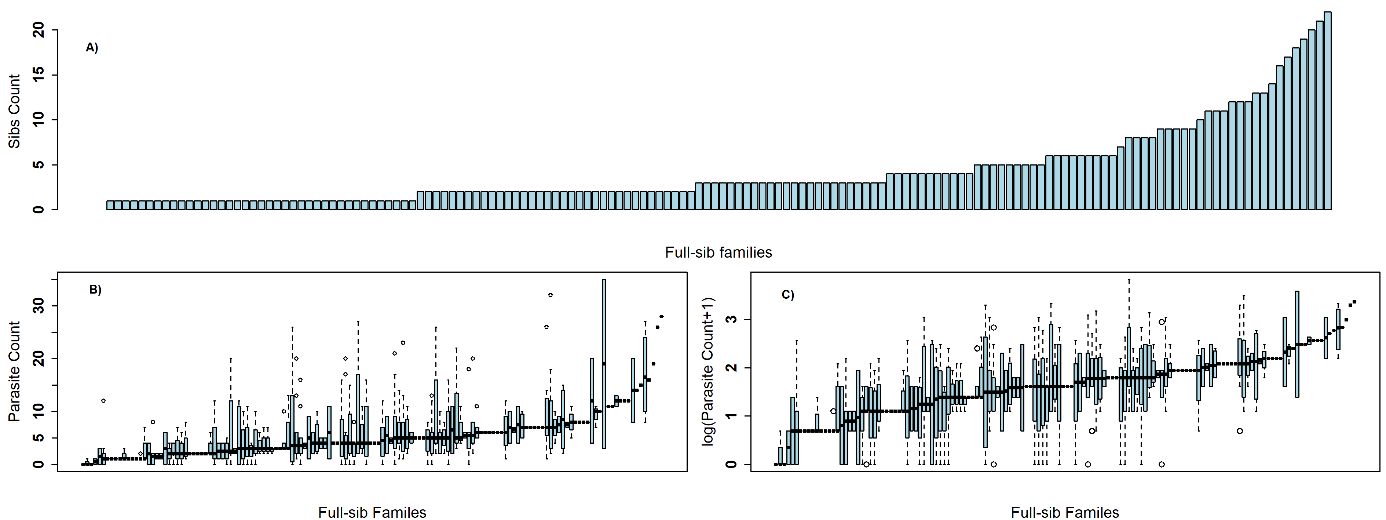


Panel **A)** is a distribution of sibs in full-fib families, panel **B)** is a distribution of parasite count across different full-sib families, and **C)** is a distribution of log transformed parasite count across different full-sib families.

**Figure S1.3:** Plot of linkage maps with distribution of markers across different linkage groups.

**Figure S1.4:** Pattern of linkage disequilibrium decay along the length (cM) of linkage groups.


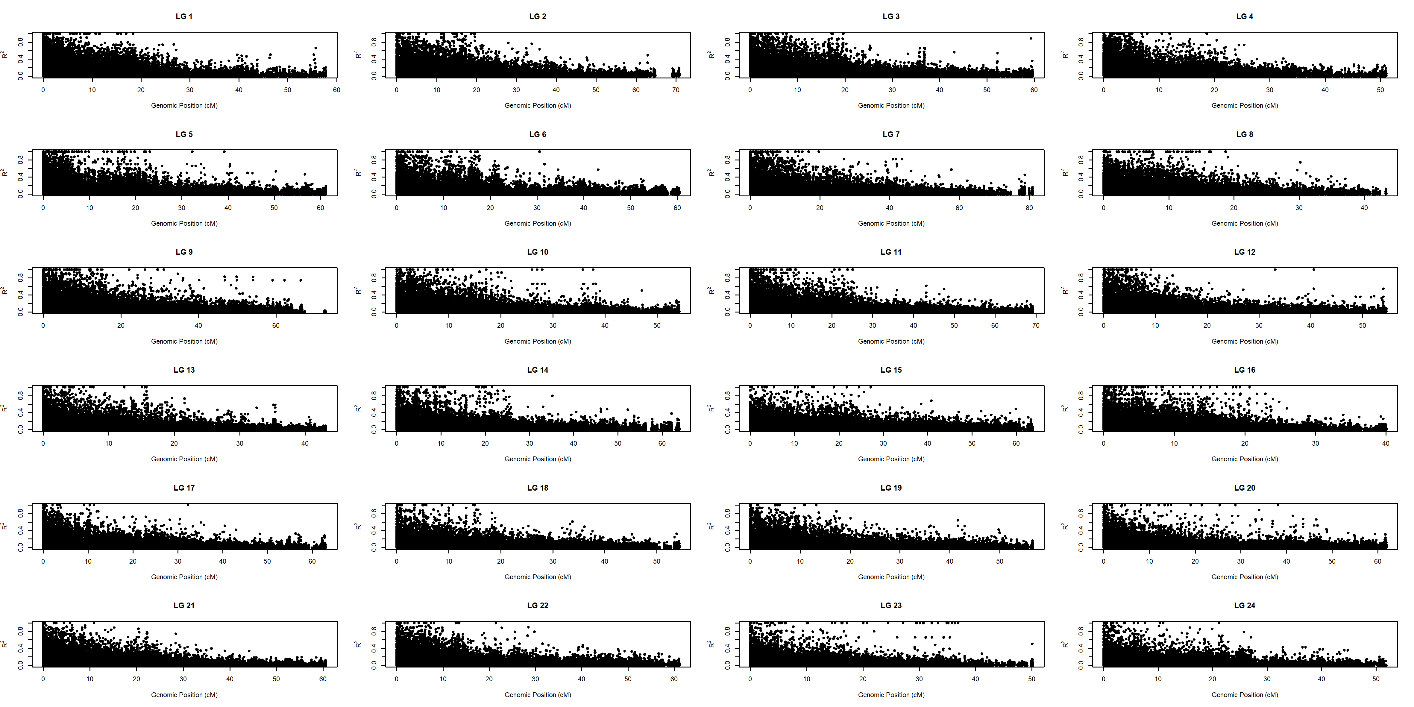


**Figure S1.5:** Heat map of Linkage disequilibrium values for the top 5 significant markers in GWAS analysis.


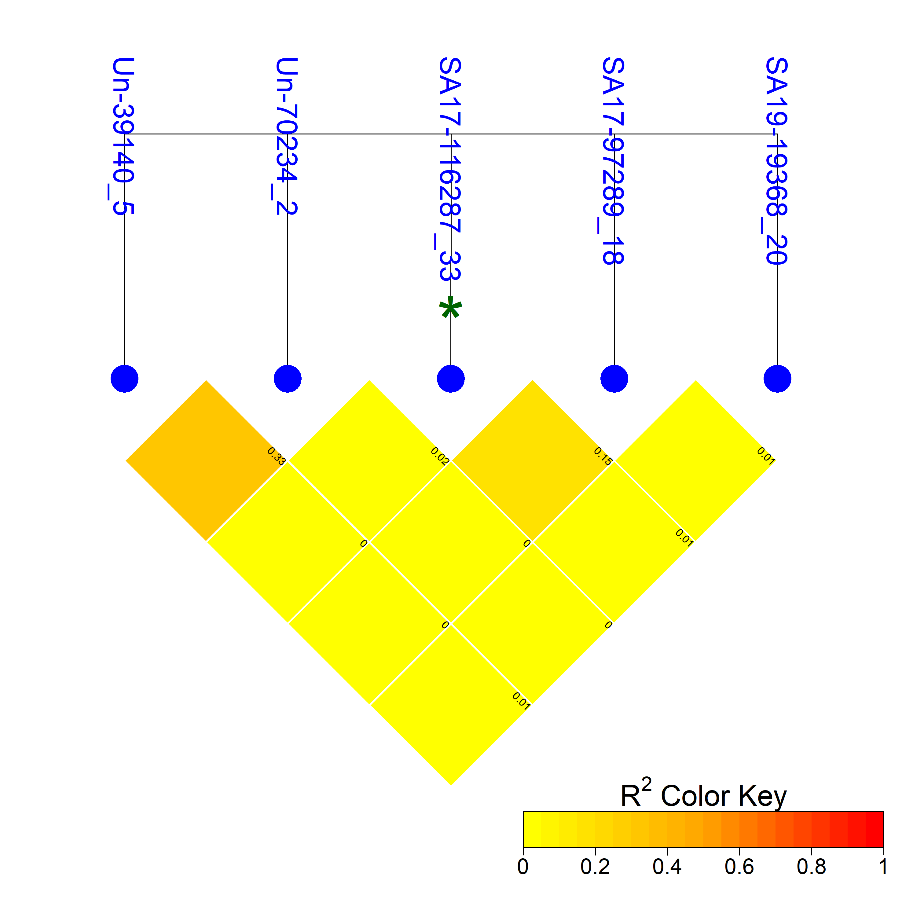


***** is a chromosome-wide significant.
